# Identifying Blood Biomarkers for Dementia Using Machine Learning Methods in the Framingham Heart Study

**DOI:** 10.3390/cells11091506

**Published:** 2022-04-30

**Authors:** Honghuang Lin, Jayandra J. Himali, Claudia L. Satizabal, Alexa S. Beiser, Daniel Levy, Emelia J. Benjamin, Mitzi M. Gonzales, Saptaparni Ghosh, Ramachandran S. Vasan, Sudha Seshadri, Emer R. McGrath

**Affiliations:** 1 The Framingham Heart Study, Framingham, MA 01701, USA; Honghuang.lin3@umassmed.edu (H.L.); himali@uthsca.edu (J.J.H.); satizabal@uthsca.edu (C.L.S.); alexab@bu.edu (A.S.B.); levyd@nhlbi.nih.gov (D.L.); emelia@bu.edu (E.J.B.); gonzalesM20@uthscsa.edu (M.M.G.); sapta@bu.edu (S.G.); vasan@bu.edu (R.S.V.); seshadri@uthscsa.edu (S.S.); 2 Department of Medicine, University of Massachusetts Medical School, Worcester, MA 01655, USA; 3 School of Public Health, Boston University, Boston, MA 02118, USA; 4 School of Medicine, Boston University, Boston, MA 02118, USA; 5 Glenn Biggs Institute for Alzheimer’s & Neurodegenerative Diseases, University of Texas Health Sciences Center, San Antonio, TX 77072, USA; 6 Population Sciences Branch, National Heart, Lung and Blood Institutes of Health, Bethesda, MD 20824, USA; 7 HRB Clinical Research Facility, National University of Ireland Galway, University Road, H91TK33 Galway, Ireland

**Keywords:** dementia, risk prediction, biomarkers, blood biomarkers, machine learning

## Abstract

Blood biomarkers for dementia have the potential to identify preclinical disease and improve participant selection for clinical trials. Machine learning is an efficient analytical strategy to simultaneously identify multiple candidate biomarkers for dementia. We aimed to identify important candidate blood biomarkers for dementia using three machine learning models. We included 1642 (mean 69 ± 6 yr, 53% women) dementia-free Framingham Offspring Cohort participants attending examination, 7 who had available blood biomarker data. We developed three machine learning models, support vector machine (SVM), eXtreme gradient boosting of decision trees (XGB), and artificial neural network (ANN), to identify candidate biomarkers for incident dementia. Over a mean 12 ± 5 yr follow-up, 243 (14.8%) participants developed dementia. In multivariable models including all 38 available biomarkers, the XGB model demonstrated the strongest predictive accuracy for incident dementia (AUC 0.74 ± 0.01), followed by ANN (AUC 0.72 ± 0.01), and SVM (AUC 0.69 ± 0.01). Stepwise feature elimination by random sampling identified a subset of the nine most highly informative biomarkers. Machine learning models confined to these nine biomarkers showed improved model predictive accuracy for dementia (XGB, AUC 0.76 ± 0.01; ANN, AUC 0.75 ± 0.004; SVM, AUC 0.73 ± 0.01). A parsimonious panel of nine candidate biomarkers were identified which showed moderately good predictive accuracy for incident dementia, although our results require external validation.

## 1. Introduction

Dementia is a significant contributor to death and dependence worldwide, with an estimated global prevalence of approximately 44 million people [1]. Early disease detection and risk prediction are key to informing the future development of effective population-level interventions for dementia prevention. Identification of dementia at the earliest preclinical or prodromal stages will offer the greatest opportunity for disease modification. Blood biomarkers for preclinical stages of dementia could also improve participant selection for phase III clinical trials. 

There is a growing appreciation that in addition to neurodegeneration, there are a number of pathways implicated in the pathophysiological changes underlying the early development of cognitive decline and dementia, with data from genome-wide association studies supporting a role for inflammation, vascular and endothelial injury, and lipid processing, amongst others [2]. A modeling approach for preclinical dementia that incorporates multiple candidate biomarkers reflecting the diverse pathophysiological pathways underlying dementia (e.g., inflammation, vascular injury, thrombosis, neurodegeneration, metabolic signaling, lipid signaling), compared to individual blood biomarkers, is likely to offer greater utility in predicting a person’s risk of dementia. Machine learning methods offer an attractive analytical strategy to this end, as one can simultaneously and efficiently evaluate multiple potential candidate biomarkers for dementia risk [3], and their potential interactions, without needing to specify *a priori* the nature (e.g., directionality or linearity) of the biomarker–outcome association. 

The objective of this study was to identify candidate circulating biomarkers for dementia at a preclinical stage, using a community-based sample of cognitively normal individuals in the Framingham Heart Study (FHS) and employing three machine-learning-based methods, namely support vector machine (SVM), eXtreme gradient boosting of decision trees (XGB), and artificial neural network (ANN). 

## 2. Materials and Methods

### 2.1. Study Sample

The FHS is a community-based, prospective cohort study initiated in 1948 that investigates risk factors and incidence of cardiovascular disease (CVD) and dementia in the community. Three generations of participants have been enrolled to date [4]. The Original Cohort was enrolled from 1948 (n = 5209) with examinations completed at 2-year intervals. From 1971, the children of the Original Cohort participants, and their spouses, were invited to enroll in the Offspring or Second Generation Cohort (n = 5124). The Offspring Cohort are examined at 4–8-year intervals, with 9 examinations completed to date. The Third Generation Cohort was enrolled from 2002 to enhance our genotypic and phenotypic understandings of CVD and other outcomes (n = 4095). This cohort consists of individuals who have an Offspring Cohort parent, with examinations occurring at 4–6-year intervals. The New Offspring Cohort was initiated in 2003 to provide additional familial data and consists of parents of Third Generation Cohort participants who had not previously been enrolled in the Offspring Cohort (n = 103). Given the greater ethnic diversity in the town of Framingham since the Original Cohort was initiated in 1948, two cohorts have since been instituted consisting of individuals of African American, Hispanic, Asian, Indian, Native American, and Pacific Islander descent. The OMNI One Cohort was formed in 1994 (n = 507) and the OMNI Two Cohort in 2003 (n = 410). The Omni Two Cohort includes some family members of OMNI One Cohort participants. Further details on FHS cohorts have been published previously [5].

For the purposes of this investigation, we included Second Generation (Offspring) Cohort participants who attended their seventh examination cycle (1998–2001, i.e., baseline for the present investigation) and who had circulating biomarkers measured at this examination, were aged 60 years or above, free of a diagnosis of dementia at baseline, and had subsequent data available on dementia status on follow-up (n = 1772). We excluded participants (n = 130) with missing data for more than half of the biomarkers under investigation and those aged < 60 years at baseline, due to the negligible number of dementia cases in our cohort prior to this age. All participants provided written informed consent, and the study was approved by the Institutional Review Board at the Boston University Medical Center. 

### 2.2. Outcome Measure

Our primary outcome measure was incident all-cause dementia occurring at any time after examination cycle seven (baseline) up to December 2016. Dementia was diagnosed in line with the Diagnostic Statistical Manual of Mental Disorders (4th edition) criteria [6]. A diagnosis of dementia was reached based on a detailed review of available neurological examination records, neuropsychological assessments, MRI brain data, outpatient and nursing home clinical records, family interview data, and any available autopsy data by a Dementia Review Committee comprising at least one neurologist and one neuropsychologist. In brief, starting from examination cycle five, all participants were systematically screened for the occurrence of new-onset dementia via yearly health status updates and the Mini-Mental State Examination (MMSE). Starting from examination cycle seven, all Offspring Cohort participants were invited to participate in neuropsychological testing (in addition to the MMSE) and a brain MRI. The comprehensive neuropsychological test battery consisted of the following components: logical memory (recognition, immediate recall, and delayed recall), a widely used measure of verbal memory and subset of the Wechsler Memory Scale (WMS); visual reproduction (recognition, immediate recall, and delayed recall), based on the WMS visual reproduction subtest; paired associate learning, a measure of ability to learn new information, a subset of the WMS; digit span (forward and backward), a measure of both working memory and simple attention, based on the Weschler Adult Intelligence Scale (WAIS); similarities, a measure of abstract reasoning, based on the WAIS; Boston naming test, a measure of naming function; trail making tests A and B, a measure of visual attention and executive function; finger tapping test, a measure of motor speed and motor dysfunction laterality; Hooper visual organization test, a measure of visuospatial function; and wide range achievement test (reading component). All tests were administered by trained examiners using standardized protocols. Further details on neuropsychological testing have previously been reported [7]. If at any point a FHS physician, a participant, or a participant’s family member is concerned about potential cognitive impairment in that participant, or if the MMSE score is less than the education-adjusted cutoff, five points lower than the participant’s prior highest score or three points lower than the preceding examination score, more in-depth cognitive testing for that participant is completed [8]. For participants with suspected cognitive impairment who do not meet diagnostic criteria for dementia, additional yearly neuropsychological testing is performed for ongoing surveillance. 

### 2.3. Baseline Characteristics

We measured baseline demographic and clinical variables at the seventh examination cycle (baseline), including age, sex, current smoking status (participant self-reported within the previous 1 year), body mass index (BMI), use of antihypertensive medication, systolic blood pressure (mean of two physician recorded measurements), history of diabetes mellitus (fasting blood glucose ≥ 7mmol/L, random blood glucose ≥ 11.1 mmol/L, or use of insulin or oral hypoglycemics), apolipoprotein E4 (ApoE4) carrier status (a carrier was defined as E2/E4, E3/E4, or E4/E4), prevalent cardiovascular disease (CVD, including peripheral vascular disease, coronary heart disease, and congestive heart failure), prevalent stroke, total cholesterol, and high-density lipoprotein cholesterol (HDL-C).

### 2.4. Measurement of Biomarkers

Circulating biomarkers were measured as part of the Markers for Vascular Cognitive Impairment and Dementia (MarkVCID) initiative [9]. At the baseline clinical visit (seventh examination cycle), fasting blood samples were obtained from the antecubital vein of participants in the supine position, immediately centrifuged, and stored at −80 °C. Plasma concentrations of each biomarker were subsequently measured from these frozen samples. We included markers of neurodegeneration (Aß42/40 ratio, clusterin), inflammation (CD14, sCD40L (also prothrombotic), GDF-15, CRP, ICAM-1, IL-6, MCP-1, MPO, OPG, P-Selectin, TNF-α, TNFR-2), thrombosis (fibrinogen, PAI-1, sCD40L), cardiac function (BNP), renal function (cystatin C), microvascular/endothelial injury (homocysteine, MMP-9), neurotrophic factors (BDNF, IGF-1, IGFBP-1,2,3 and VEGF), adipokines (adiponectin, leptin, resistin), lipids (APO-A1, APO-B, HDL-C, TC), hormone measures (FGF23), vitamins (vitamin B12, vitamin D), and metabolic factors (HbA1c, insulin) (Table 1). Biomarkers were measured using the optimal assay available, including Quanterix Single Molecule Array (Simoa, Norway), ELISA, Luminex and Meso Scale Discovery (MSD). Plasma total tau (T-tau) was measured at examination cycle eight and analyzed using Quanterix Simoa assay. All assays were performed in duplicate with excellent inter-assay coefficients of variation. Performance characteristics of individual biomarker assays are provided in Table 1. 

### 2.5. Statistical Analysis

Biomarkers were inverse logarithmically transformed to approximate a normal distribution and to facilitate cross comparisons between biomarkers. We excluded biomarkers with missing data in more than half of participants (n = 5). For biomarkers with <50% missing data, we performed multiple imputation using the chained equations approach to impute missing values [10]. Baseline characteristics were compared between those with and without incident dementia during follow-up, using the Wilcoxon Rank-Sum test for continuous variables and Fisher’s exact test for categorical variables. *p* < 0.05 was considered significant.

We evaluated three different machine learning methods in the current study, including support vector machine, eXtreme gradient boosting of decision trees, and artificial neural network. Support vector machine (SVM) involves constructing a hyperplane that separates two different classes of feature vectors with a maximum margin; one class represents cases and the other represents controls [11]. The eXtreme gradient boosting of decision trees (XGB) is a tree boosting method with superior performance [12]. An algorithm integrates many decision trees to improve predictive performance and new models are added to correct existing model errors at each step. An artificial neural network (ANN) mimics a human neuron network, with an input layer of neurons representing the descriptors in the training set, and a summation layer of neuron outputs to obtain the estimated probability density function for that class of neurons.

In our base machine learning models, adjusted for age, sex, survival time, and ApoE E4 carrier status, we included all 38 available biomarkers. 

We subsequently implemented stepwise feature elimination to remove any noninformative biomarkers from the models (i.e., biomarkers with *p* > 0.05). One thousand imputations were performed, and biomarkers that were consistently identified as significant in more than 90% of permutations were selected as the most informative biomarkers. We then evaluated the performance of the machine learning models using five-fold cross-validation. In each cycle, four-fifths of samples was used to build the training model, and the remaining one-fifth of samples was used for testing the model. This process was repeated five times until all samples were used for testing once. Given that a different threshold cutoff could result in a different number of positive and negative predictions, we used the receiver operating characteristic (ROC) curve to summarize model predictive performance. In addition, we calculated the specificity, precision, and overall accuracy using different sensitivity cutoffs (presented in Supplemental Table S1). To further validate the robustness of our analysis, we performed 1000 permutations. Results are presented as means ± standard deviations (SD). 

### 2.6. Sensitivity Analyses

The role of tau in the pathophysiology of Alzheimer’s disease (AD) and other dementias is well recognized. As plasma t-tau was not measured at examination cycle seven, we did not include it in our primary analysis. However, we completed a sensitivity analysis including plasma t-tau measured at examination cycle eight (2004–2011), to determine if addition of t-tau to the machine learning models of 38 plasma biomarkers resulted in improved model performance. For the purposes of this analysis, we included those individuals who were dementia-free, aged 60 years or above at the time of examination cycle seven, attended examination cycle eight, had data available on circulating t-tau at examination cycle 8 and the remaining panel of 38 biomarkers at examination cycle seven, and who had data available on dementia status on follow-up (n = 1159). 

In an additional sensitivity analysis, we built a predictive model using logistic regression analysis and compared the predictive performance of this model (AUC) with the three machine learning models. All analyses were conducted using R statistical software v4.0.3. 

## 3. Results

### 3.1. Cohort Descriptives

The current investigation included 1642 eligible participants from the Offspring Cohort (mean age 69 ± 6 years, 52.7% women) who attended the seventh examination cycle. Descriptive characteristics of the participants are presented in Table 2. Participants were followed for a mean (SD) of 12 ± 5 years during which 243 (14.8%) were diagnosed with incident dementia. Individuals who developed dementia were more likely to be female, older, have a lower Mini-Mental State Examination (MMSE) score, have a higher baseline systolic blood pressure, and a history of cardiovascular disease and diabetes mellitus.

### 3.2. Biomarkers Predictive of Dementia

**Figure 1:**
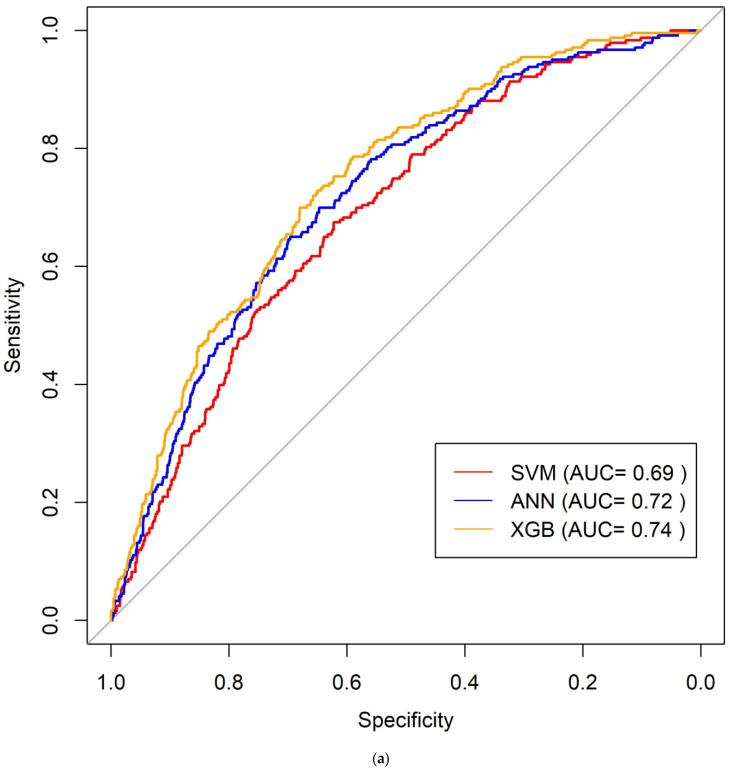

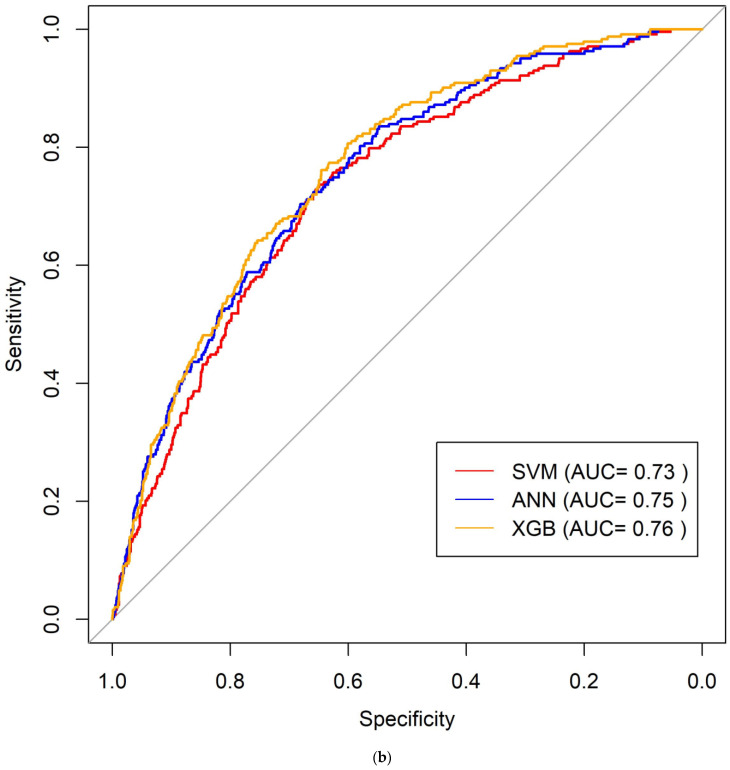
(**a**) Prediction of dementia using 38 biomarkers. (**b**) Prediction of dementia using the 9 most informative biomarkers.

In our sample, 43 plasma biomarkers were measured at examination cycle seven, of which 38 were available in more than half of the participants and eligible for inclusion in our machine learning models. Of the three machine learning models evaluated, XGB showed the strongest predictive accuracy for incident dementia (AUC = 0.74 ± 0.01), followed by ANN (AUC 0.72 ± 0.01), and SVM (AUC 0.69 ± 0.01) (Figure 1a).

Using stepwise-elimination-based feature selection, we identified a subset of nine highly informative biomarkers for predicting dementia risk that were consistently selected in more than 90% of permutations (Table 1). When we rebuilt the machine learning models using these nine biomarkers, all three updated models showed improved predictive performance: XGB demonstrated the highest predictive accuracy (AUC 0.76 ± 0.01), followed by ANN (AUC 0.75 ± 0.004), and SVM (AUC 0.73 ± 0.01) (Figure 1b). In a sensitivity analysis, addition of plasma tau to the machine learning models of these nine biomarkers resulted in no appreciable change in the AUC: XGB (AUC 0.76 ± 0.01), ANN (AUC 0.75 ± 0.004), and SVM (AUC 0.72 ± 0.01). In a second sensitivity analysis using logistic regression modeling, the logistic regression model did not perform better (AUC 0.72 ± 0.01) compared to the three machine learning models. 

## 4. Discussion

In this community-based cohort, we identified important candidate biomarkers for dementia risk prediction incorporating a panel of 38 potential candidate markers and utilizing three different machine learning methods. The XGB model demonstrated the strongest predictive accuracy for incident dementia. A parsimonious subset of nine candidate biomarkers were identified, which together provided the highest predictive accuracy for dementia risk, demonstrating moderately good accuracy. 

In our investigation, XGB and ANN showed moderate predictive accuracy for incident dementia with an AUC of greater than 0.70 in models incorporating all 38 available biomarkers. Using stepwise feature elimination, we identified a subset of nine most informative biomarkers for inclusion in a more parsimonious model. In analyses confined to these nine biomarkers, all three machine learning models showed improved predictive accuracy (AUC of 0.76 for XGB), likely due to the reduced noise-to-signal ratio when only the most informative biomarkers were included.

A number of studies have proposed various blood multimarker panels for the screening and diagnosis of dementia and for predicting conversion from mild cognitive impairment (MCI) to dementia [13,14,15,16,17,18,19,20,21,22,23,24,25,26,27,28,29,30,31,32,33]. A study of machine learning approaches in patients with MCI identified increased levels of plasma AB42, t-tau, and p-tau181 in those with AD dementia compared to MCI and controls [34]. Indeed, many of the biomarkers identified in our sample overlap with those included in previous panels, e.g., Aß42/40 [14,35], TNF-α [13,15,16,18,31], PAI-I [16], leptin [16], IGFBP-2 [28,31,33], MCP-1 [32], and homocysteine [28]. While many prior studies have shown promising diagnostic accuracy in differentiating individuals with dementia from normal controls, subsequent validation attempts have proved challenging, which has limited the development of multimarker panels to date [20,36,37,38]. Promisingly, a recent study replicated 6 of 13 identified protein biomarkers in an external cohort [33]. Our study adds to the existing literature, by identifying a multimarker panel of nine promising candidate markers in cognitively normal individuals at baseline in a community-based setting.

The majority of the biomarkers we identified in this investigation have previously been associated with cognitive functioning and/or dementia. Aß42/40 has been established as a promising blood biomarker for dementia screening [39,40,41]. In addition, we identified two inflammatory biomarkers associated with incident dementia, tumor necrosis factor-α (TNF-α) and monocyte chemotactic protein-1 (MCP-1). TNF-α is thought to play a role in the development of ß-amyloid and tau pathology [42], with some early phase clinical trial data suggesting a potential cognitive benefit of TNF-α inhibitors in patients with AD dementia [43,44]. MCP-1, a member of the chemokine family and a marker of glial cell activation, has been associated with ß-amyloid pathology in murine models of AD dementia [45]. Elevated levels of MCP-1 have also been associated with amnestic difficulties and lower medial temporal lobe volumes in patients with mild cognitive impairment and AD dementia [46]. We also identified a number of vascular and metabolic markers associated with dementia, many of which have previously been associated with dementia, cognitive impairment, or dementia brain pathology, including homocysteine [47], plasminogen activator inhibitor-1 (PAI-1) [48], cystatin C [49], and leptin [50,51], although results for leptin have been conflicting [52]. High-density lipoprotein cholesterol (HDL-C) was also identified as one of the most informative biomarkers in our analysis. A previous study reported an association between elevated levels of high-density lipoprotein cholesterol (HDL-C) and reduced dementia risk (after adjusting for ApoE E4 genotype) [53], although a subsequent meta-analysis failed to detect an association [54]. In addition, our machine learning models identified insulin-like growth factor-binding protein-2 (IGFBP-2) (a neurotrophic factor thought to inhibit the neuroprotective effects of the insulin-like growth factor signaling system in the brain) as an important predictor of dementia risk, consistent with findings from prior studies [28,33,55]. 

Our study has some important limitations. Our sample size was modest; however, we included carefully phenotyped individuals who were closely followed for the development of incident dementia over a relatively long duration using stringent surveillance criteria. We were unable to account for time to event in our machine learning analyses and thus we cannot comment on the predictive value of this panel of biomarkers for time to risk of dementia. Our goal was instead to utilize machine learning approaches to identify a core set of potential biomarkers implicated in the complex biological pathways underpinning dementia. The participants included in this study were exclusively adults of European descent. Thus, our findings may not be generalizable to other ethnicities/age groups. Biomarker data were missing for a number of participants, requiring use of multiple imputation. However, the proportion of individuals with missing biomarker data was relatively small for the majority of included biomarkers (data for 28 (75%) biomarkers were missing in <5% of participants). We did not have data available on other identified markers of dementia at examination cycle seven to include in our analyses, e.g., phosphorylated tau species such as p-tau181 and p-tau217, neurofilament light chain (NFL), or glial fibrillary acidic protein (GFAP). However, the addition of tau (measured at examination cycle eight) to our models did not materially alter the predictive performance of the models. Admittedly, total-tau is a less specific biomarker for AD dementia compared to phosphorylated tau species. Our study does not establish a causal relationship between individual biomarkers and risk of incident dementia. Our diagnosis of dementia was based on clinical criteria rather than biomarker-based definitions (e.g., amyloid or tau deposition using cerebrospinal fluid analysis or brain positron emission tomography imaging), although our approach is more consistent with routine clinical practice in which CSF and PET data are not readily available. Finally, our results will require external validation in other cohorts (e.g., community-based cohorts in other countries and clinic-based cohorts of patients presenting with minor cognitive symptoms), as well as those with greater representation across other ethnicities.

## 5. Conclusions

Machine learning is an efficient strategy to predict the synergistic effects of multiple biomarkers which might be related to dementia in a nonlinear way. In our community-based cohort, our three machine learning models showed moderately good predictive accuracy in identifying individuals at high risk of developing dementia, with XGB demonstrating the greatest predictive accuracy. A parsimonious subset of nine biomarkers showed promise in predicting dementia in cognitively healthy adults in a community setting, although our results will require replication in other cohorts.

## Figures and Tables

**Table 1:** List of blood-based biomarkers with assay performance characteristics.

Variable	Min	25% Quantile	Median	75% Quantile	Max	Missing Rate (%)	Inter-Assay CV (%)	Intra-Assay CV (%)	LLOQ
Aß42/40 *	0.1	0.2	0.3	0.3	1.2	1	-	-	-
Adiponectin (ug/mL)	0.9	5.9	9	14.1	59.9	16.8	9.6	6.2	0.08
ApoA1 (pg/mL)	8.2 × 10^7^	7.8 × 10^8^	9.1 × 10^8^	1.0 × 10^9^	3.3 × 10^9^	0.7	7.3	11.8	3.4 × 10^5^
ApoB (pg/mL)	2.4 × 10^8^	6.1 × 10^8^	7.1 × 10^8^	8.4 × 10^8^	3.6 × 10^9^	0.7	13.4	6.6	2.6 × 10^6^
BDNF (pg/mL)	1713.7	1.7 × 10^4^	2.3 × 10^4^	2.9 × 10^4^	5.6 × 10^4^	8.2	7.6	4.8	312
BNP (pg/mL)	10.1	132	275	548.8	8530	1	7.2	10.3	9.7
CD14 (pg/mL)	7.66 × 10^6^	1.44 × 10^7^	1.65 × 10^7^	1.92 × 10^7^	4.24 × 10^7^	0.3	14.5	3.6	5.8 × 10^4^
CD40L (ng/mL)	0.1	0.5	1.1	3.3	29.5	0.2	14.1	4.9	0.005
Clusterin (pg/mL)	1.54 × 10^6^	4.44 × 10^7^	5.07 × 10^7^	5.88 × 10^7^	1.23 × 10^8^	0.4	12.6	9.1	1.5 × 10^4^
CRP mg/L	0.2	1.2	2.6	5.5	99.8	0.2	5.3	3.2	0.2
Cystatin C * (mg/L)	0.6	0.9	1	1.1	7	1.6	3.3	2.4	0.3
FGF-23 (pg/mL)	19	56	69	89	434	13.6	13.4	5.5	18.7
Fibrinogen (mg/dL)	204	343	387	436	763	0.4	4.4	1.1	90
GDF-15 (pg/mL)	249	602	770	1030	2.1 × 10^4^	0.3	2.9	2.3	40
HbA1c (%)	1.7	5.2	5.6	6.1	14.6	10.1	<2.5	<2.5	0
HDL-C * (mg/dL)	17	40	50.5	63	136	0.1	2.8	0.9	17
Homocysteine * (umol/L)	3.3	6.9	8.4	10.3	84.3	0.1	7	4.5	3.2
ICAM-1 (ng/mL)	29	217	247.1	290	1327.5	0.1	6	3.9	<0.4
IGF-1 (ng/mL)	24.3	84.8	105.1	129.6	377.3	8.9	4.5	3.4	23.5
IGFBP-1 (pg/mL)	1000	5545	1.0 × 10^4^	2.0 × 10^4^	1.7 × 10^5^	2.4	5.4	2.5	979
IGFBP-2 * (pg/mL)	1.79 × 10^6^	8.24 × 10^6^	1.22 × 10^7^	1.77 × 10^7^	9.31 × 10^7^	0.8	8.7	6	1.6 × 10^6^
IGFBP-3 (pg/mL)	9.4 × 10^4^	1.8 × 10^5^	2.2 × 10^5^	2.6 × 10^5^	6.2 × 10^5^	0.5	18	4.4	272
IL-6 (pg/mL)	0.6	2.1	3.2	4.8	104.4	0.4	9	3.7	<0.7
Insulin (pmol/L)	14.9	59.2	80.4	111.6	1296	1.2	6.1	3.9	12
Leptin * (pg/mL)	413	2290	5425	11875	129000	5.9	7	3.2	397
MCP-1 * (pg/mL)	34.5	267.1	328.4	398.3	2139.8	2	11.1	3.8	5.7
MMP-9 (pg/mL)	1.7 × 10^4^	3.8 × 10^4^	4.8 × 10^4^	6.4 × 10^4^	6.1 × 10^5^	0.5	10	3.9	243
MPO (ng/mL)	4.9	27.4	38.9	58.7	332.1	3.1	NR	3.2	0.2
OPG (pmol/L)	0.6	5	6	7.1	26.9	0.3	NR	3.7	0.1
PAI-1 * (pg/mL)	4600	1.4 × 10^4^	1.9 × 10^4^	2.6 × 10^4^	1.2 × 10^5^	0.5	10.8	3.6	449
P-selectin (ng/mL)	2.5	29.4	37.3	46.8	194.9	0.2	NR	3.2	<0.5
Resistin (ng/dL)	1.2	10.3	13.2	17.3	110	16.2	11	4.6	0.2
TC (mg/dL)	83	174	197	219	357	0	1.5	0.7	20
TNF-a * (pg/dL)	0.3	1	1.3	1.7	20.9	22.7	11.3	7.6	0.1
TNFR-2 (pg/mL)	681.6	1814.8	2170.1	2665.2	8383.4	2.4	NR	2.3	0.2
VEGF (pg/mL)	15.3	162.9	288.6	459.7	1728.4	8.4	14.7	4.3	9.2
Vitamin B12 (pg/mL)	54.7	323.1	411.2	522.9	2931.2	0.1	10	8.5	34.6
Vitamin D (ng/mL)	3.1	14.8	19.4	24.2	58.5	45.8	8.5	NR	2.2
T-tau (pg/mL)	0.8	3.3	4.1	5	17	30.6	7.5	4.1	0.01

* The nine most informative biomarkers are highlighted in bold. Abbreviations: LLOQ, lower limit of quantification; CV, coefficient of variation; NR = not reported (for some biomarkers, no inter-assay CV is reported as assays were run at the same time/using the same plate); Aß42/40, ß-amyloid 42/ß-amyloid 40; Apo A1, Apolipoprotein A-1; ApoB, Apolipoprotein B; BDNF, brain-derived neurotrophic factor; BNP, brain natriuretic peptide; CD14, monocyte differentiation antigen; CD40L, cluster of differentiation 40 ligand; CRP, C-reactive protein; FGF23, fibroblast growth factor 23; GDF-15, growth differentiation factor 15; HbA1c, glycosylated hemoglobin; HDL-C, high-density lipoprotein cholesterol; ICAM-1, intercellular cell-adhesion molecule-1; IGF-1, insulin-like growth factor 1, pg/mL; IGFBP, insulin-like growth factor-binding protein; IL-6, interleukin-6; MCP-1, monocyte chemotactic protein-1; MMP-9, matrix metallopeptidase 9; MPO, myeloperoxidase; OPG, osteoprotegerin; PAI-1, plasminogen activator inhibitor 1; TC, total cholesterol; TNF-α; tumor necrosis factor-α; TNFR-2, tumor necrosis factor receptor-2;VEGF, vascular endothelial growth factor; T-tau, total tau.

**Table 2:** Baseline descriptives.

	Dementia (n = 243)	No Dementia (n = 1399)	*p* Value
Women	144 (59.3)	721 (51.5)	0.03
Age, years	72 ± 6	68 ± 6	<0.001
MMSE (median, IQR)	28 (27–29)	29 (28–30)	<0.001
Current smoker	22 (9.1)	118 (8.4)	0.75
Body mass index (BMI), kg/m^2^	27.6 ± 5.0	28.1 ± 5.1	0.14
Total cholesterol, mg/dL	195 ± 38	199 ± 36	0.11
HDL cholesterol, mg/dL	52 ± 17	53 ± 17	0.25
Prevalent CVD	62 (25.5)	250 (17.9)	0.005
Prevalent stroke	17 (7.0)	57 (4.1)	0.06
Hypertension treatment	125 (51.4)	599 (42.8)	0.01
Diabetes	14 (5.8)	151 (10.8)	0.01
Systolic blood pressure, mmHg	136± 20	132 ± 20	0.001

Baseline characteristics were measured at examination cycle 7 and are presented separately for individuals who subsequently developed dementia during follow-up, and those who remained dementia-free. Values are reported as mean ± standard deviation for continuous variables and n (%) for categorical variables.

## Data Availability

The de-identified data used in these analyses can be obtained from the NHLBI database and the NCBI dbGaP.

## Supplementary Materials

The following supporting information can be downloaded at: https://www.mdpi.com/article/10.3390/cells11091506/s1, Table S1: Performance of machine learning models under different sensitivity cutoffs.
