# Supplementary material for: Identifying Blood Biomarkers for Dementia Using Machine Learning Methods in the Framingham Heart Study
Source: Cells. 2022 Apr 30;11(9):1506. doi: 10.3390/cells11091506 (PMC9100323; doi:10.3390/cells11091506)
Supplement: Supplementary file 1 [file cells-11-01506-s001.zip › cells-1666994-supplementary.pdf]

Table S1. Performance of machine learning models under different sensitivity cutoffs

Sensitivity	SVM			ANN			XGB		
	**Specificity**	**Precision**	**Accuracy**	**Specificity**	**Precision**	**Accuracy**	**Specificity**	**Precision**	**Accuracy**
10.0%	97.1%	37.8%	84.2%	97.7%	43.2%	84.7%	97.5%	41.0%	84.5%
25.0%	91.4%	33.4%	81.5%	94.6%	44.4%	84.3%	92.7%	37.3%	82.7%
50.0%	78.7%	29.0%	74.5%	82.1%	32.7%	77.4%	82.5%	33.2%	77.7%
75.0%	62.1%	25.6%	64.0%	60.0%	24.6%	62.3%	64.8%	27.0%	66.3%
90.0%	37.2%	19.9%	45.0%	37.9%	20.1%	45.6%	40.9%	20.9%	48.2%
